# Homology of the head sensory structures between Heterotardigrada and Eutardigrada supported in a new species of water bear (Ramazzottiidae: *Ramazzottius*)

**DOI:** 10.1186/s40851-023-00221-w

**Published:** 2023-11-27

**Authors:** Ji-Hoon Kihm, Krzysztof Zawierucha, Hyun Soo Rho, Tae-Yoon S. Park

**Affiliations:** 1 Division of Earth Sciences, Korea Polar Research Institute, 26 Songdomirae-ro, Yeonsu-gu, Incheon, 21990 Korea; 2 Department of Animal Taxonomy and Ecology, Faculty of Biology, Adam Mickiewicz University, Uniwersytetu Poznańskiego 6, Poznań, 61-614 Poland; 3 East Sea Environmental Research Center, East Sea Research Institute, Korea Institute of Ocean Science & Technology, 48 Haeyanggwahak-gil, Uljin, Gyeongsangbuk-do 36315 Korea; 4 Polar Sciences, University of Science and Technology, 217 Gajeong-ro, Yuseong-gu, Daejeon, 34113 Korea

**Keywords:** Cephalic sensory structure, Arctic, Ancestral character, Morphogroup, Greenland

## Abstract

Phylum Tardigrada is represented by microscopic eight-legged panarthropods that inhabit terrestrial and marine environments. Although tardigrades are emerging model animals for areas of research including physiology, evolutionary biology, and astrobiology, knowledge of their external morphology remains insufficient. For instance, homologies between marine and terrestrial relatives largely remain unexplored. In the present study we provide detailed pictures of the head sensory organs in a new tardigrade, *Ramazzottius groenlandensis* sp. nov. Specimens were collected from a mixed moss and lichen sample on Ella Island, East Greenland. The new species differs from congeneric species in the presence of polygonal sculpturing on the dorsal cuticle, which is accentuated in the posterior region of the body, a lateral papilla on leg IV, and distinctive egg morphology. A Bayesian phylogenetic analysis (18S rRNA + 28S rRNA + COI) places the new species within the genus *Ramazzottius* with high confidence. Interestingly, the new species shows a full set of well-developed cephalic organs, which correspond to all sensory fields found in eutardigrades. Details on the full set of head organs were present only for heterotardigrades. The surface of these organs is covered with small pores, which presumably play a sensory role. This discovery suggests the homology of head sensory structures between heterotardigrades and eutardigrades, implying that the distinctive arrangement and positioning of sensory organs on the head is a plesiomorphic feature of tardigrades. Moreover, we find that the *Ramazzottius oberhaeuseri* morphotype forms a morphogroup, not a monophyletic species complex.

**Supplementary Information:**

The online version contains supplementary material available at 10.1186/s40851-023-00221-w.

## Background

 Tardigrades (also known as water bears) are eight-legged microscopic metazoans that form a separate phylum included in the Panarthropoda. These animals are important members of the meiofaunal community [1] and live in a wide variety of habitats from aquatic to terrestrial environments, from deep seas to high mountains. They can be found in sediments, soil, bryophytes, lichens, and even on glaciers [2–4]. Thanks to their ability to enter into a latent, ametabolic life stage—cryptobiosis—some tardigrades can survive extreme conditions such as low and high temperatures, high irradiation, or pressure extremes [5–7]. Due to their resistance and simple body plan, some tardigrades, such as *Hypsibius exemplaris* Gąsiorek, Stec, Morek & Michalczyk, 2018 [8] and *Ramazzottius varieornatus* Bertolani & Kinchin, 1993 [9] have become renowend model organisms [10–13]. However, the evolution and links between their morphological traits, as well as their ancestral states, remain unclear (however, see Fig. 2.2 in [14]), and the homology of inter-specifically variable morphologies is underexplored.

To date, approximately 1,500 species of tardigrades have been described worldwide [15]. These are grouped into two classes: Eutardigrada and Heterotardigrada. The two groups differ in their external morphology and evolutionary history [16]. Heterotardigrades live in both terrestrial and marine ecosystems and possess several pairs of sensory organs on the head, such as cirri and clavae. For instance, echiniscoideans have ten head sensory organs, including a pair of internal/external/lateral cirri and a pair of primary/secondary clavae, while marine heterotardigrades [17] have up to 13 sensory organs, including a pair of internal/external/lateral cirri, a pair of primary/secondary/tertiary clavae, and an unpaired median cirrus [18, 19]. In contrast, sensory organs are significantly reduced or completely absent in eutardigrades. Although several neuroanatomical studies have revealed that some representatives of eutardigrades have sensory fields that may be homologous to the head sensory organs of heterotardigrades [20–23], whether these possibly homologous sensory organs appear on the surface of eutardigrades remains unresolved. Recently, a neuroanatomical comparative study involving *Echiniscus testudo* (Doyére, 1840) [24] and *Hypsibius exemplaris* [8] suggested possible homology between the cephalic sensory fields of the eutardigrades and the cephalic sensory organs of heterotardigrades [20].

Homology, defined as “the possession by two or more species of a trait derived, with or without modification, from their common ancestor” [25], is a central concept in understanding the evolution of morphological traits [26]. As an evolutionarily traceable marker of certain lineages, morphological homology forms the basis for phylogenetic reconstruction [27]. The position (anatomical location) and the structure of a character are essential criteria for detecting morphological homology [28]. However, many tardigrade species have been observed and illustrated exclusively by light microscopy, which has hindered integrative understanding of details of certain morphological features. For example, head structures, such as elliptical organs [29–31], frontal lobes [32, 33], and cephalic papillae [21, 32, 34] are, in a small number of eutardigrades, reminiscent of the sensory organs of heterotardigrades. However, in some eutardigrade species these structures are usually detectable only in SEM [35–37], and are rarely observable under the light microscopy. Observation of tardigrades under SEM could thus yield information on the detailed surface morphological features and provide insights into homologies between marine heterotardigrades and limnoterrestrial eutardigrades.

*Ramazzottius* Pilato & Binda, 1986 is a genus of limnoterrestrial eutardigrades occurring worldwide, including polar regions and high mountains [35]. *Ramazzottius* species are characterised by the presence of apophyses for the insertion of the stylet muscles (AISM) in the shape of blunt-hooks and articulated external claws. Some species have paired elliptical organs on the head [38–40]. Representatives of *Ramazzottius* are considered xerophilic, often exposed to sunlight, and can be found in substrata such as bryophytes and lichens [41]. The vast Arctic tundra in Greenland, with its abundant cryptogams, thus forms a highly suitable habitat for tardigrades. However, of the twenty-nine valid *Ramazzottius* species [15], only two species—*R. montivagus* (Dastych, 1983) [42] and *R. oberhaeuseri* (Doyère, 1840) [24]—have been documented from Greenland [43]. Particularly, *R. oberhaeuseri*, one of the earliest described tardigrade species, is the only *Ramazzottius* species reported from East Greenland [44]. Moreover, because *R. oberhaeuseri* was established with insufficient morphological and morphometric data, doubts have persisted regarding its taxonomic validity [31]. A recent integrative study even revealed that several species exist under the name *R. oberhaeuseri*, forming the *Ramazzottius oberhaeuseri* complex [31].

Here we provide an integrative description of a new species *R. groenlandensis* sp. nov. from Ella Island, East Greenland, with partial molecular sequences of three genes (a small ribosome subunit (18S rRNA), a large ribosome subunit (28S rRNA), and cytochrome oxidase c subunit I (COI)). Notably, SEM images of this new species show a set of head sensory organs that are most likely homologous to the sensory organs of heterotardigrades.

## Materials & methods

### Sample processing

During the 2019 summer season, the KOPRI (Korea Polar Research Institute) palaeontology team collected a sample of mixed bryophytes and lichens from limestone near Lake September (72°50'51.23"N, 25°5'8.71"W, 471 m above sea level (a.s.l.)) (Supplementary Fig. 1). The dry sample was kept in a plastic bag, brought to KOPRI (Incheon, Korea), and stored at 4ºC for two months. Subsequently, the sample was placed on a dish filled with Volvic® water and was squeezed over a Petri dish. Tardigrades were retrieved from the supernatant under a stereomicroscope (Leica M205C).

### Microscopy and imaging

For light microscopic observation, specimens were prepared following a previously reported method [45]. Tardigrades were relaxed at 60ºC for 30 min and were mounted on a microscope slide in Hoyer’s medium. Subsequently, the slides were dried seven days at 60ºC, sealed with nail polish, and examined under a differential interference contrast (DIC) microscope (Carl Zeiss Axio Imager 2), with the camera AxioCam HRc.

For SEM observation, specimens were prepared following a previously reported method [46]. First, the tardigrades were incubated at 60ºC for 30 min. After fixation in 4% formaldehyde solution, individuals were washed three times with distilled water. Afterwards, specimens were subjected to a water/ethanol series and an ethanol/hexamethyldisilazane (HMDS) series subsequently, with 10% increasing concentration at 10-min intervals (from 10 to 100%), following a previously reported method [47]. Buccal-pharyngeal apparatuses were collected after tardigrades discarded them during molting. Dried animals and buccal-pharyngeal apparatuses were then mounted on SEM stubs using an eyebrow and coated with a thin layer of gold. SEM observations were made using a field emission SEM JSM-7200F, at KOPRI.

### Morphometrics

Selection of characters for the morphometry and the morphological terminology follow those of the previous references [31, 39]. All measurements are given in micrometers (µm) and were conducted under the DIC microscope. Characters were measured when the specimens were mounted in a suitable orientation on the slide. Body length was measured from the anterior tip to the posterior end of the body, excluding the legs IV. The *pt* index is the percent ratio of the length of a character to the length of buccal tube [48]. For measurements of claws, the scheme described in [49] as adapted by [31] was used.

For species identification and differentiation, original descriptions and redescriptions were used [9, 31, 35, 36, 38, 50–61].

### Genotyping

DNA was extracted from six individuals using QIAamp DNA Micro Kit. A PCR mixture was prepared with a total volume 25 µl, containing 12.5 µl Takara EmeraldAmp® PCR Master Mix, 2 µl of DNA template, 0.25 µl of each primer and 10 µl of *dd*H_2_O. Three DNA fragments were sequenced, namely, the small ribosome subunit (18S rRNA), the large ribosome subunit (28S rRNA) and the cytochrome oxidase c subunit I (COI). The PCR settings followed those described in a previous method [31]; primers and original references for PCR settings of all partial genes are listed in Supplementary Tables 1 & 2. The PCR products were sent to a commercial company for sequencing (Cosmogenetech, Korea). The sequences were processed in Geneious v. 9.0.5 (https://www.geneious.com) and submitted to GenBank.

### Genetic distance and phylogenetics

Phylogenetic analysis was conducted using concatenated 18S rRNA + 28S rRNA + COI sequences of Ramazzottiidae that belong to fourteen taxa, with *Hypsibius convergens* (Urbanowicz, 1925) [62] as the outgroup. For the concatenated data set, we selected taxa for which at least two sequences among 18S, 28S, and COI were available in NCBI. We used fragments of sequences of specific species, originating from single specimens (vouchers) or specimens identified recently by using integrative approach, in order to prevent possible confusion arising from taxonomic misidentification. Sequences were downloaded from GenBank, a full list of accession numbers is given in Supplementary Data 1. 18S rRNA and 28S rRNA sequences were aligned using the Q-INS-i method, and COI sequences were aligned using G-INS-1 in MAFFT online service [63] and checked manually in BioEdit v. 7.0.5.3 [64]. The sequences were concatenated in the following order: 18S rRNA, 28S rRNA, and COI.

Partitionfinder v. 2.1.1 [65], under Bayesian Information Criterion (BIC), was used to find the best scheme of partitioning and substitution models. The following models were suggested: TRNEF+I for 18S rRNA, TRN+I for 28S rRNA, and TIM+I, TRN+G, TRNEF+G for the first, second, and third codon positions of COI, respectively. Bayesian inference (BI) posterior probabilities (PP) were calculated using MrBayes v. 3.2.6 [66]. Two random starting trees, each of four Metropolis coupled Markov chains Monte Carlo method, were launched for 3 × 10^7^ generations. Trees were sampled every 1,000 generations and the initial 10% trees were discarded as burn-in. Convergence was assessed by checking the standard MrBayes convergence diagnostics: estimated sample size scores > 200, average standard deviation of split frequencies values < 0.01, and potential scale reduction factor values ~ 1.00. Obtained tree samples were summarized as a majority rule consensus tree. The final consensus tree was visualized using FigTree v. 1.4.4.

Additionally, we conducted another phylogenetic analysis based on COI. We included all available *Ramazzottius* COI sequences in the dataset (EF620418 and KU900021 were excluded due to short sequence length.). Fifty-three sequences, including *R. groenlandensis* sp. nov., were analyzed. The methods of alignment, model search and phylogenetic analysis are identical to those described above. The suggested model from Partitionfinder v.2.1.1 was TRN+I for the first, and TRN+G for second, and TRNEF+G for third codon positions of COI, respectively.

Pairwise distances between nucleotide sequences were calculated using a distance model for all codon positions, as implemented in MEGA X [67]. *p*-distance calculations for all positions containing gaps and missing data were eliminated. The analysis of COI involved 53 *Ramazzottius* sequences, and the analyses of 18 and 28S rRNA involved eleven and eight nucleotide sequences, respectively (including one sequence of *R. groenlandensis* sp. nov. with other ramazzottiids), and the final dataset had sequences with lengths of 448 (COI), 820 (18S) and 738 (28S), respectively. Using data sets for COI, we performed a genetic species delimitation analyses by Automatic Partitioning (ASAP [68]). Analyses were performed on https://bioinfo.mnhn.fr/abi/public/asap/asapweb.html with default settings. The results of these analyses are given in Supplementary Data 3.

## Results

### Taxonomic account

Phylum: Tardigrada Doyère, 1840 [24].

Class: Eutardigrada Richters, 1926 [69].

Order: Parachela Schuster et al., 1980 [70].

Superfamily: Hypsibioidea Pilato, 1969 [71] (in Marley et al., 2011 [39]).

Family: Ramazzottiidae Sands et al., 2008 [72].

Genus: *Ramazzottius* Binda and Pilato, 1986 [73].

*Ramazzottius groenlandensis* sp. nov.

Synonyms: *Ramazzottius* cf. *rupeus* in [37], *Ramazzottius* cf. *oberhaeuseri* species 2 [31].

urn:lsid:zoobank.org:act:2964B209-9AE6-477B-9626-9AE28342B8C0.

### Examined material

Fifty-eight animals and three eggs on slides in Hoyer’s medium (2 eggs were ruptured during preparation), 181 animals and three eggs mounted on stubs for SEM observations.

### Type repositories

The holotype (slide code: KOPRIF 2019-Ella-Rama 01), 52 paratype specimens (slide codes: KOPRIF 2019-Ella-Rama 02–53), three egg specimens (slide codes: KOPRIF 2019-Ella-Rama Egg 01–03), and 12 SEM stubs including 181 animal specimens and three egg specimens (stub codes: KOPRIF 2019-SEM-Ella-Rama 01–12) were deposited in the KOPRI Paleontology collection (Division of Earth Sciences, KOPRI, Korea); five paratypes (slide codes: KOPRIF 2019-Ella-Rama 54–58), were deposited at the Department of Animal Taxonomy and Ecology at Adam Mickiewicz University, Poznań, Poland.

### Type locality

72°50'51.23"N, 25°5'8.71"W, 471 m a.s.l.: the limestone deposit near Lake September, Ella Island, Greenland.

### Etymology

The name *groenlandensis* refers to the locality, Greenland, where the species was formally identified and described.

### General description

**Fig. 1:**
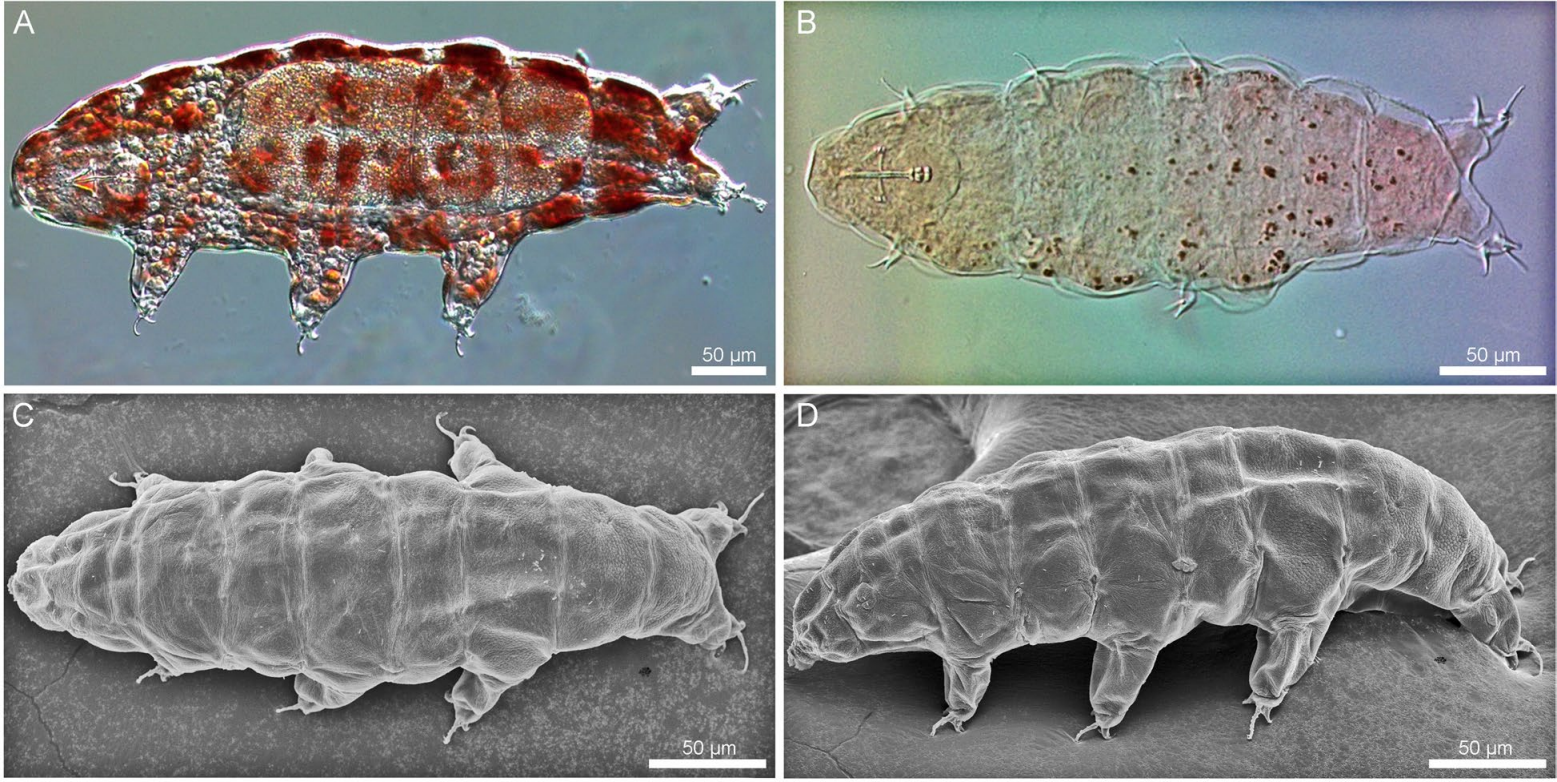
*Ramazzottius groenlandensis* sp. nov. Differential interference contrast microscope (DIC) images and SEM images: **A**, **B** DIC images; **C**, **D** SEM images. **A** a living specimen. **B** the holotype. **C**, **D** dorsal view and oblique lateral view

**Fig. 2:**
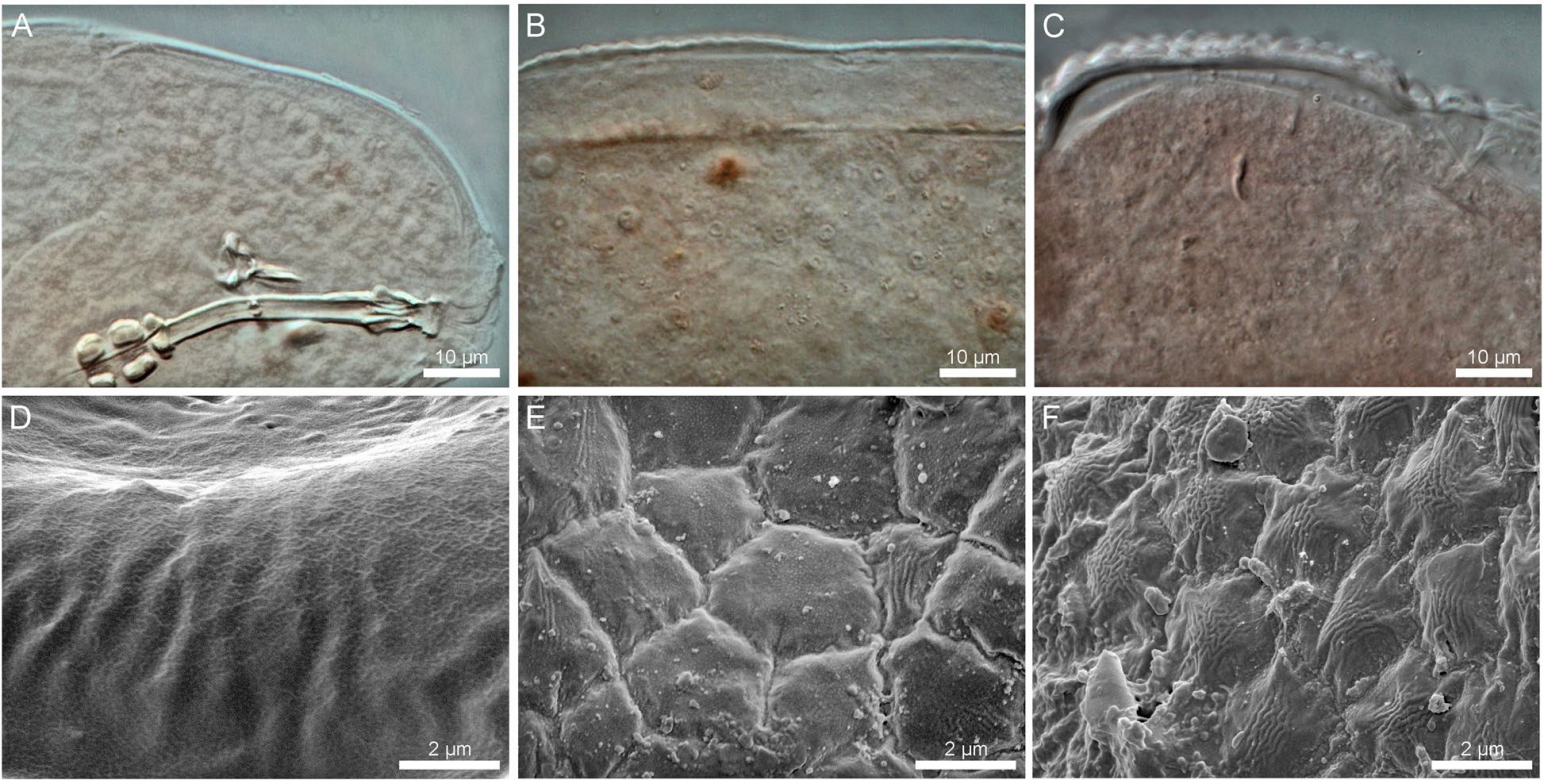
The cuticular sculpturing of *Ramazzottius groenlandensis* sp. nov. Differential interference contrast microscope (DIC) images and SEM images: **A**–**C** DIC images; **D**–**F** SEM images. **A**, **D** cuticular surface of the head region. **B**, **E** cuticular surface of the middle part of the trunk. **C**, **F** cuticular surface of the posterior part of the trunk

**Fig. 3:**
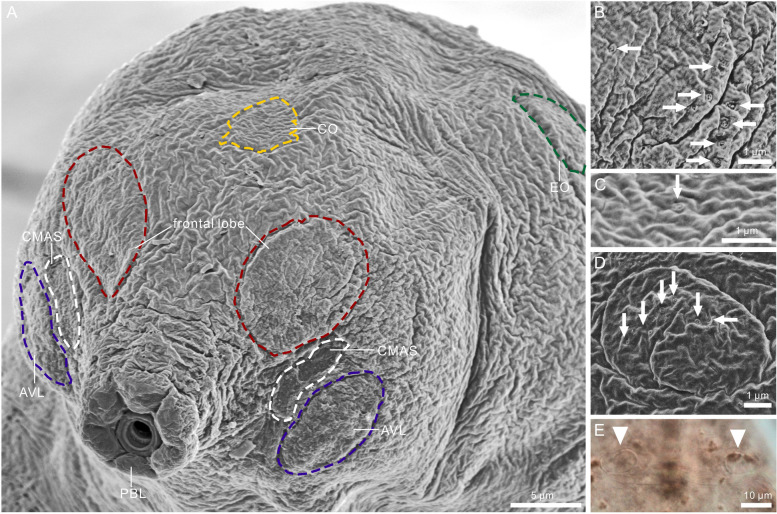
Head sensory organs of *Ramazzottius groenlandensis* sp. nov. Differential interference contrast microscope (DIC) images and SEM images: **A**–**D** SEM images; **E** DIC images. **A** head region. **B** frontal lobe. **C** centrodorsal organ (CO). **D**, **E** elliptical organ (EO). Arrows and arrow head indicate pores and EO, respectively. AVL: anteroventral lobe; CMAS: cribriform muscle attachment site; CO: centrodorsal organ; EO: elliptical organ; PBL: peribuccal lobe

**Fig. 4:**
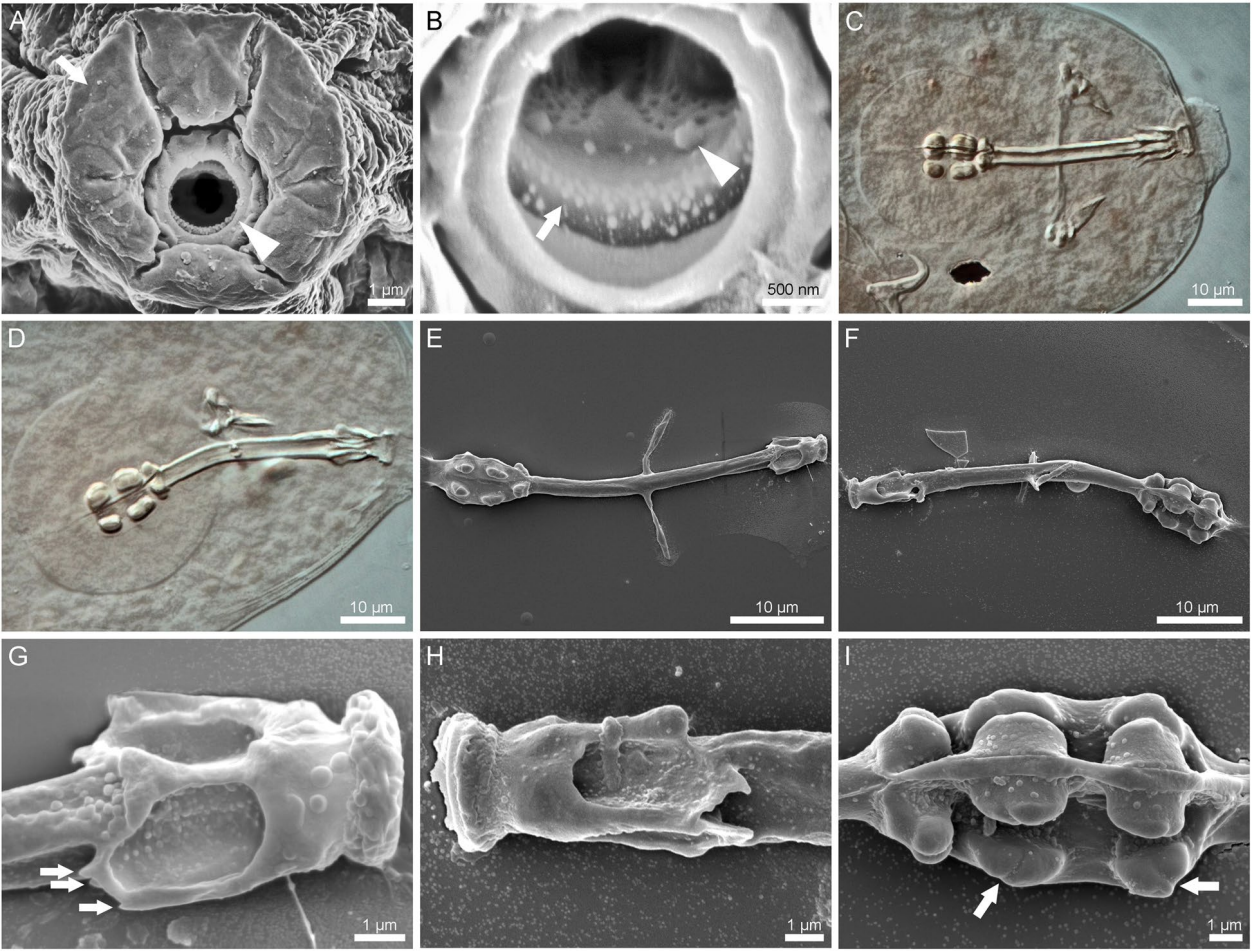
The mouth opening and the buccal-pharyngeal apparatus of *Ramazzottius groenlandensis* sp. nov. Differential interference contrast microscope (DIC) images and SEM images: **A**, **B**, **E**–**I** SEM images; **C**, **D** DIC images. **A** anterior view of the mouth and the peribuccal lobes. Arrow and arrowhead indicate the peribuccal lobe and the mouth opening, respectively. **B** oral cavity armature with two bands of teeth and perforated area. Arrow and arrowhead indicate the first band and the second band of teeth, respectively. **C** ventral view of the buccal-pharyngeal apparatus. **D** oblique lateral view of the buccal-pharyngeal apparatus. **E** ventral view of the buccal-pharyngeal apparatus. **F** lateral view of the buccal-pharyngeal apparatus. **G** ventral view of the apophysis for the insertion of the stylet muscles (AISM). Arrows indicate the posterior tips of AISM. **H** oblique lateral view of the AISM. **I** placoids. Arrows indicate placoid constrictions

**Fig. 5:**
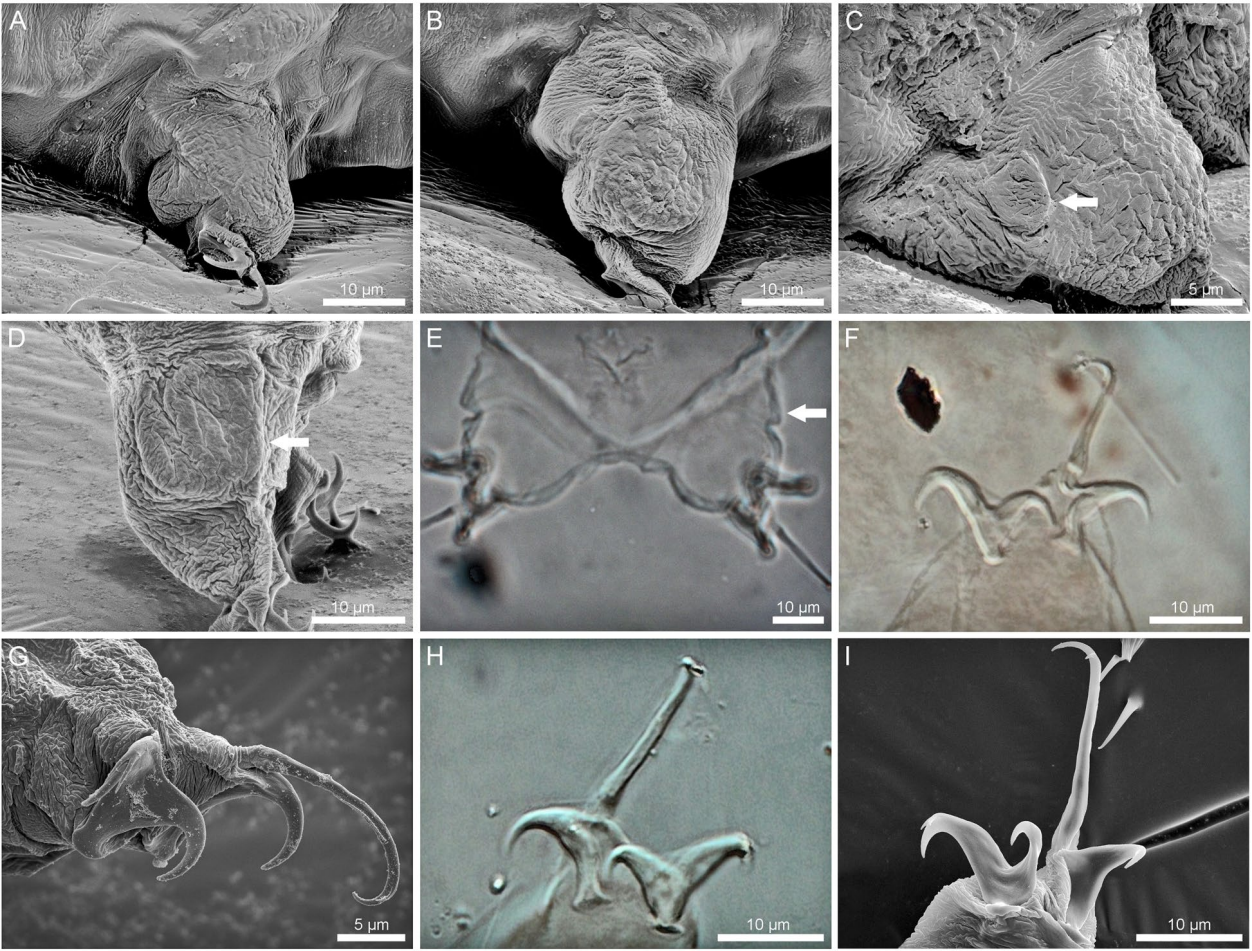
Legs and claws of *Ramazzottius groenlandensis* sp. nov. Differential interference contrast microscope (DIC) images and SEM images: **A**–**D**, **G**, **I** SEM images; **E**, **F**, **H** DIC images. **A** leg I **B** leg II **C**–**E** leg IV. Arrows indicate papilla on leg IV. **F** claw I. **G** claw II. **H**–**I** claw IV

Figs. 1, 2, 3, 4 and 5; measurement and basic statistics in Table 1; raw data in Supplementary Data 4.

Body color varied from red to brown. The pigmented surface divided by transparent transversal stripes, which disappear after mounting in Hoyer’s medium (Fig. 1A, B). Eyes absent in live animals. Dorsal cuticular sculpturing present (Fig. 1C, D), while the ventral cuticle is smooth. The anterior part of the body smooth or covered by irregular wrinkles on head (Fig. 2A, D), flat and weak polygonal sculptures in the middle region of the body (Fig. 2B, E), and strongly marked, tubercle-like structures in the caudal region of the body (Fig. 2C, F). More posteriorly, the cuticle sculpturing is larger. Under SEM, seven sensory organ-like structures present on the head (Fig. 3A): a pair of frontal lobes, a pair of anteroventral lobes (AVL), a pair of elliptical organs (EO), and a centrodorsal organ (CO). On the anterolateral sides of the head, two pairs of structures present above (frontal lobes, Fig. 3A) and below (AVL, Fig. 3A) the cribriform muscle attachment sites (CMAS, Fig. 3A), respectively. On the surface of the frontal lobes several micropores are scattered (Fig. 3B). Additionally, there is a small region which is likely to show slightly different cuticular surface on the anterodorsal part of the head (CO, Fig. 3A). This region has a pore at the centre (Fig. 3C). Two EO (Fig. 3A) with several pores present on the dorsoposterior part of the head (Fig. 3D). Only EO visible under the light microscopy (Fig. 3E).

Mouth opening anteroventral (sub-terminal). Mouth surrounded by six peribuccal lobes (Fig. 4A). Peribuccal lamellae and peribuccal papulae absent. The oral cavity possesses two bands of teeth (Fig. 4B) that are poorly visible in DIC micrographs (Fig. 4C–D). The first band consists of small triangular teeth arranged in several rows on the ring fold. The second band, composed of cone shaped-teeth in a single row, occurs behind the first band. A perforated area is present behind the second band of teeth (Fig. 4B). Buccal-pharyngeal apparatus of the *Ramazzottius*-type (Fig. 4C–I) [58]; i.e. asymmetrical apophysis for the insertion of the stylet muscles (AISM) with slightly longer ventral apophysis (Fig. 4D, H) and both apophyses with caudal apices. AISM has posterior tips on each lateral side (Fig. 4G) (although it is worth noting that there is a possibility that the middle tip in Fig. 4G is an artefact). The buccal tube bent ventrally after the stylet support insertion point (Fig. 4D, F). The pharyngeal bulb spherical to oval, with triangular apophyses and two macroplacoids, all clearly separated. Macroplacoids roundish; the 1^st^ macroplacoid slightly longer than the 2^nd^ macroplacoid. A small constriction in both 1^st^ and 2^nd^ macroplacoids visible (Fig. 4C, I). Microplacoid absent.

While cuticle on leg I is smooth (Fig. 5A), legs II–IV exhibit polygonal sculpturing (Fig. 5B, C). On the lateral side of leg IV, a papilla (= a gibbosity in [53]) is present (see remarks). The papilla on the leg IV varies in size (Fig. 5C–E); from a quarter to more than half of the length of the leg when the leg is fully extended (Fig. 5D). Claws of the *Ramazzottius*-type (Fig. 5F–I), i.e., two claws of the same leg extremely different from each other in size and shape. Primary branches of external claws and posterior claws longer than the primary branches of internal claws and anterior claws. The bases of all claws have a smooth pseudolunule. Primary branches of external/posterior claws with cuticular flexible portions, connected to the secondary branches (“*oberhaeuseri* group” claw in [58]). Accessory points present on all primary branches.

**Table 1:** Measurements (in µm) of selected morphological characters of *Ramazzottius groenlandensis* sp. nov. mounted in Hoyer’s medium

		Range						Mean		SD	
Character	N	µm			*pt*			µm	*pt*	µm	*pt*
Body length	55	166.9	‒	370.3	*520.9*	‒	*1164.0*	289.8	*911.6*	53.8	*144.3*
**Buccal-pharyngeal tube**											
Buccal tube length	58	24.3	‒	37.5				31.6		2.5	
Stylet support insertion point	57	14.1	‒	21.6	*51.0*	‒	*63.2*	18.5	*58.4*	1.6	*2.9*
Buccal tube external width	58	1.8	‒	3.7	*5.9*	‒	*12.4*	2.7	*8.4*	0.4	*1.2*
Buccal tube internal width	58	0.9	‒	2.1	*2.9*	‒	*7.0*	1.4	*4.3*	0.3	*0.9*
**Placoid lengths**											
Macroplacoid 1	57	2.8	‒	4.9	*9.2*	‒	*14.7*	3.7	*11.8*	0.5	*1.5*
Macroplacoid 2	57	2.4	‒	4.3	*7.0*	‒	*13.8*	3.3	*10.6*	0.5	*1.6*
Macroplacoid row	57	6.6	‒	10.1	*20.2*	‒	*33.4*	8.2	*26.0*	0.9	*2.8*
**Claw I lengths**											
External base	58	5.7	‒	11.4	*19.7*	‒	*35.6*	8.3	*26.4*	1.4	*4.0*
External primary branch	57	7.7	‒	16.9	*29.3*	‒	*51.6*	12.9	*40.7*	1.7	*5.0*
External secondary branch	52	4.7	‒	9.9	*14.9*	‒	*31.6*	7.2	*22.9*	1.2	*3.8*
Internal base	58	3.9	‒	8.9	*12.6*	‒	*28.9*	6.3	*19.9*	1.2	*3.4*
Internal primary branch	58	5.1	‒	11.0	*19.3*	‒	*34.2*	8.3	*26.2*	1.3	*3.5*
Internal secondary branch	55	3.7	‒	8.7	*14.0*	‒	*27.7*	6.4	*20.4*	1.1	*3.1*
**Claw II lengths**											
External base	55	5.9	‒	12.6	*18.3*	‒	*37.2*	8.9	*28.2*	1.6	*4.6*
External primary branch	55	7.9	‒	17.9	*32.3*	‒	*53.3*	14.5	*45.6*	1.8	*4.4*
External secondary branch	54	3.5	‒	10.1	*14.3*	‒	*31.2*	7.6	*24.0*	1.5	*4.2*
Internal base	55	4.5	‒	11.0	*12.6*	‒	*34.4*	6.5	*20.4*	1.2	*3.4*
Internal primary branch	55	5.8	‒	14.3	*20.1*	‒	*44.6*	9.0	*28.3*	1.4	*4.0*
Internal secondary branch	53	4.7	‒	10.0	*17.9*	‒	*29.7*	7.4	*23.2*	1.0	*2.8*
**Claw III lengths**											
External base	55	5.3	‒	12.6	*15.0*	‒	*39.3*	9.3	*29.4*	1.6	*4.9*
External primary branch	54	11.9	‒	20.9	*39.7*	‒	*57.9*	15.6	*48.7*	1.8	*4.6*
External secondary branch	49	4.5	‒	11.1	*15.0*	‒	*34.6*	8.1	*25.4*	1.5	*4.5*
Internal base	54	4.6	‒	9.3	*14.4*	‒	*29.5*	6.6	*20.7*	1.1	*3.1*
Internal primary branch	54	6.5	‒	11.4	*21.6*	‒	*36.1*	9.2	*28.8*	1.2	*3.3*
Internal secondary branch	52	5.2	‒	10.0	*16.8*	‒	*30.2*	7.4	*23.3*	1.0	*2.7*
**Claw IV lengths**											
Anterior base	56	5.1	‒	8.8	*16.7*	‒	*26.6*	7.0	*22.2*	0.9	*2.7*
Anterior primary branch	56	6.5	‒	12.1	*19.6*	‒	*38.4*	9.7	*30.6*	1.2	*3.4*
Anterior secondary branch	53	4.4	‒	10.0	*17.7*	‒	*32.9*	7.7	*24.3*	1.3	*3.8*
Posterior base	57	7.1	‒	13.0	*21.7*	‒	*40.9*	9.7	*30.8*	1.3	*4.0*
Posterior primary branch	56	11.0	‒	20.5	*43.5*	‒	*61.0*	17.4	*54.9*	1.7	*3.9*
Posterior secondary branch	52	4.6	‒	10.2	*14.9*	‒	*32.5*	7.6	*24.2*	1.3	*4.1*

*Abbreviations: N *Number of specimens, *pt *Percent ratio of the length of a character to the length of buccal tube, *SD* Standard deviation

### Eggs

**Fig. 6:**
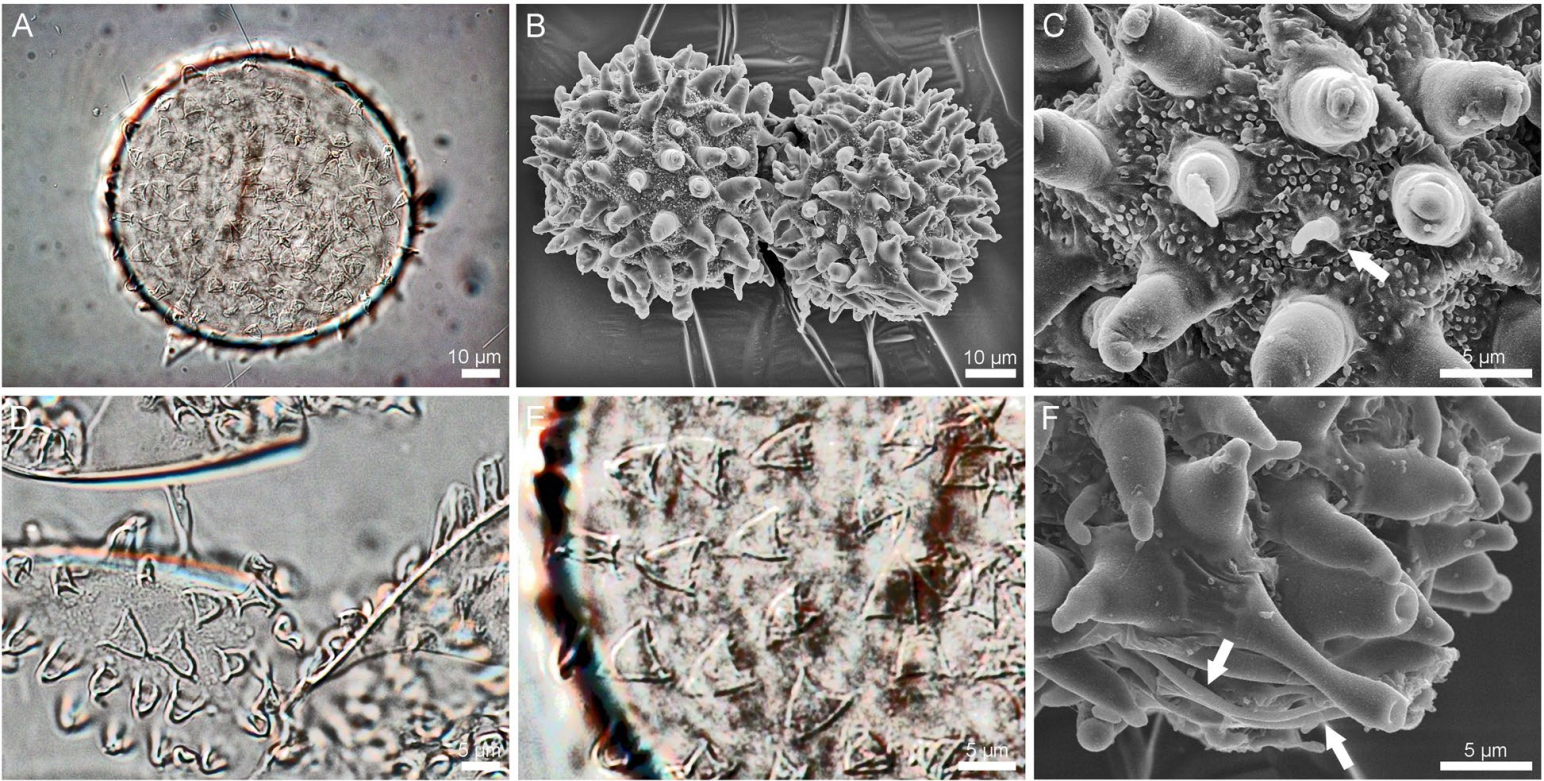
Eggs of *Ramazzottius groenlandensis* sp. nov. Differential interference contrast microscope (DIC) images and SEM images: **A**, **D**, **E** DIC images; **B**, **C**, **F** SEM images. **A** a whole egg. **B** two eggs. **C** granulated surface of the egg chorion. **D**–**E** variable morphology of processes. **F** processes with a concave tip. Arrows indicate filamentous processes

Fig. 6; measurement and basic statistics in Table 2; raw data in Supplementary Data 4.

Laid free, white, spherical (Fig. 6A, B). Chorion surface between processes granulated (Fig. 6C, D). Processes show various morphology and shape (Fig. 6C–F): i.e., most processes cone-shaped whereas other processes filamentous. While most processes have bulbous tips, some have concave tips (Fig. 6F).

**Table 2:** Measurements (in µm) of selected morphological characters of *Ramazzottius groenlandensis* sp. nov. egg. N, the number of specimens, SD, standard deviation

Character	N	Range	Average	SD
Bare diameter (µm)	1	80.6		
Full diameter (µm)	1	92.4		
Process height (µm)	45	3.5 ‒ 11.4	6.2	1.7
Process base width (µm)	45	1.9 ‒ 7.0	4.6	1.7
Process base/height ratio (%)	45	32.1 ‒ 149.4	78.5	28.6
Number of processes on the egg circumference	1	30		

### Morphological differential diagnosis

*Ramazzottius groenlandensis* sp. nov. is characterized by the presence of dorsally sculptured cuticle, several head sensory organs, two macroplacoids with constrictions and the presence of pseudolunules under the claws. The egg of *Ramazzottius groenlandensis* sp. nov. is characterized by processes with variable morphology and granulated chorion surface. *R. groenlandensis* sp. nov. differs specifically from

- *Ramazzottius affinis* Bertolani, Guidetti & Rebecchi, 1994 [50] known from Monte Serra Santa, Italy (1260 m a.s.l.), found in lichen from limestone by: the presence of accessory points on external and internal primary claw branches, the lack of a thicker buccal tube wall at the stylet support insertion point (SSIP), and the *pt* indices of the primary branches of claw II and IV, i.e. external primary branch of claw II (*78.26–80.41* in *R. affinis* vs. *32.3–52.3* in *R. groenlandensis* sp. nov.) and posterior primary branch of claw IV (*78.54–85.18* in *R. affinis* vs. *43.5–61.0* in *R. groenlandensis *sp. nov.);
- *Ramazzottius bunikowskae* Kaczmarek, Michalczyk & Diduszko, 2006 [38] known from lichens in Olkhon Island at Lake Baikal by: the presence of the sculpturing on legs, and the different oral cavity armature (one band in *R. bunikowskae* vs. two bands in *R. groenlandensis* sp. nov.);
- *Ramazzottius libycus* Pilato, D’Urso & Lisi, 2013 [54] known from mosses in Libya by: the shape of processes (hemispherical in *R. libycus* vs. conical or filamentous in *R. groenlandensis* sp. nov.);
- *Ramazzottius littoreus* Fontoura, Rubal & Veiga, 2017 [55] known from supralittoral lichens in Spain and Portugal by: the lack of the polygonal sculpturing on the head and the leg I;
- *Ramazzottius nivalis *Dastych, 2006 [56] known from lichens in the Alps (3707 m a.s.l.) by: the lack of a particularly long basal flexible unit in external claws of *R. groenlandensis*, different *pt* index of the posterior primary branch of claw IV (*66.6–78.6* in *R. nivalis* vs. *43.45–61.02* in *R. groenlandensis* sp. nov.) and the presence of granules on the chorion (absent in *R. nivalis* vs. present in *R. groenlandensis* sp. nov.);
- *Ramazzottius oberhaeuseri* known from mosses in France by: the cuticular sculpture of the caudo-dorsal body region (smooth or weak in *R. oberhaeuseri* vs. robust and intense in *R. groenlandensis* sp. nov.) and the shape of egg processes (hemispherical in *R. oberhaeuseri* vs. conical or filamentous in *R. groenlandensis* sp. nov.);
- *Ramazzottius rupeus* Biserov, 1999 [57] known from lichens in Novaya Zemlya by: sculpturing on the head (lack of sculpturing of the head in *R. groenlandensis*), the *pt* index of the posterior primary branch of claw IV (*76.5* ± *3.0* in *R. rupeus* vs. *43.5–61.0* in *R. groenlandensis* sp. nov.) and the diameter of egg with processes (67.0–79.0 μm in *R. rupeus* vs. 92.4 μm in *R. groenlandensis* sp. nov.);.
- *Ramazzottius sabatiniae* Guidetti, Massa, Bertolani, Rebecchi & Cesari, 2019 [58] known from Starr Nunatak, Victoria Land, Antarctica, found in mosses in soil by: egg surface (smooth in *R. sabatiniae* vs. granulated in *R. groenlandensis* sp. nov.).

### Genetic comparison

A GenBank search using BLAST algorithm and our sequence data indicated that the COI sequence of *Ramazzottius groenlandensis* sp. nov. is most similar to that of *R.* cf. *rupeus* deposited by [37] (GenBank accession number: MG432810). 18S rRNA sequence of *R. groenlandensis* sp. nov. is the most similar to that of *Ramazzottius varieornatus* (GenBank accession number: AP013352 [6]). 28S rRNA sequence of *R. groenlandensis* sp. nov. is most similar to those of *Ramazzottius* sp. DE.002 (GenBank accession number: MG432817 [37]), and *R. varieornatus* (GenBank accession number: AP013352 [6]).

Length of COI partial sequence was trimmed to 658 bp (GenBank: OR596527) and the 18S rRNA sequence was trimmed to 1721 bp (GenBank: OR600266), while the 28S sequence was trimmed to 784 bp long (GenBank: OR600265). The COI sequence of *Ramazzottius groenlandensis* sp. nov. differs by one base pair of the COI sequence of *R.* cf. *rupeus* (Genbank accession number: MG432810) from Northern Svalbard, and by two base pairs different to that of *R.* cf. *oberhaeuseri* (Genbank accession numbers: EU251381, EU251382 [74]) from Northern Apennines, Italy.

The intraspecific and interspecific ranges of *p*-distances within the 53 *Ramazzottius* sequences (COI) are 0–3.3% and 12.5–22.8%, respectively. Interspecific ranges of *p*-distance within eight ramazzottiid species for 18S and six ramazzottiid species for 28S are as follows for 18S: 0.4–3.1%, and 28S: 1.6–6.7%.

### ASAP

The ASAP analysis of 53 COI sequences (including all *Ramazzottius* COI sequences available from NCBI) identified fourteen putative species at asap score = 2.5 (*Ramazzottius* cf. *rupeus* (MG432810) [37], *Ramazzottius* cf. *oberhaeuseri* species 2 (EU251381–2) [31] are *R. groenlandensis* sp. nov. Other sequences are clearly different from other taxa in *Ramazzottius*, see Supplementary Data 3).

### Phylogenetic analyses

**Fig. 7:**
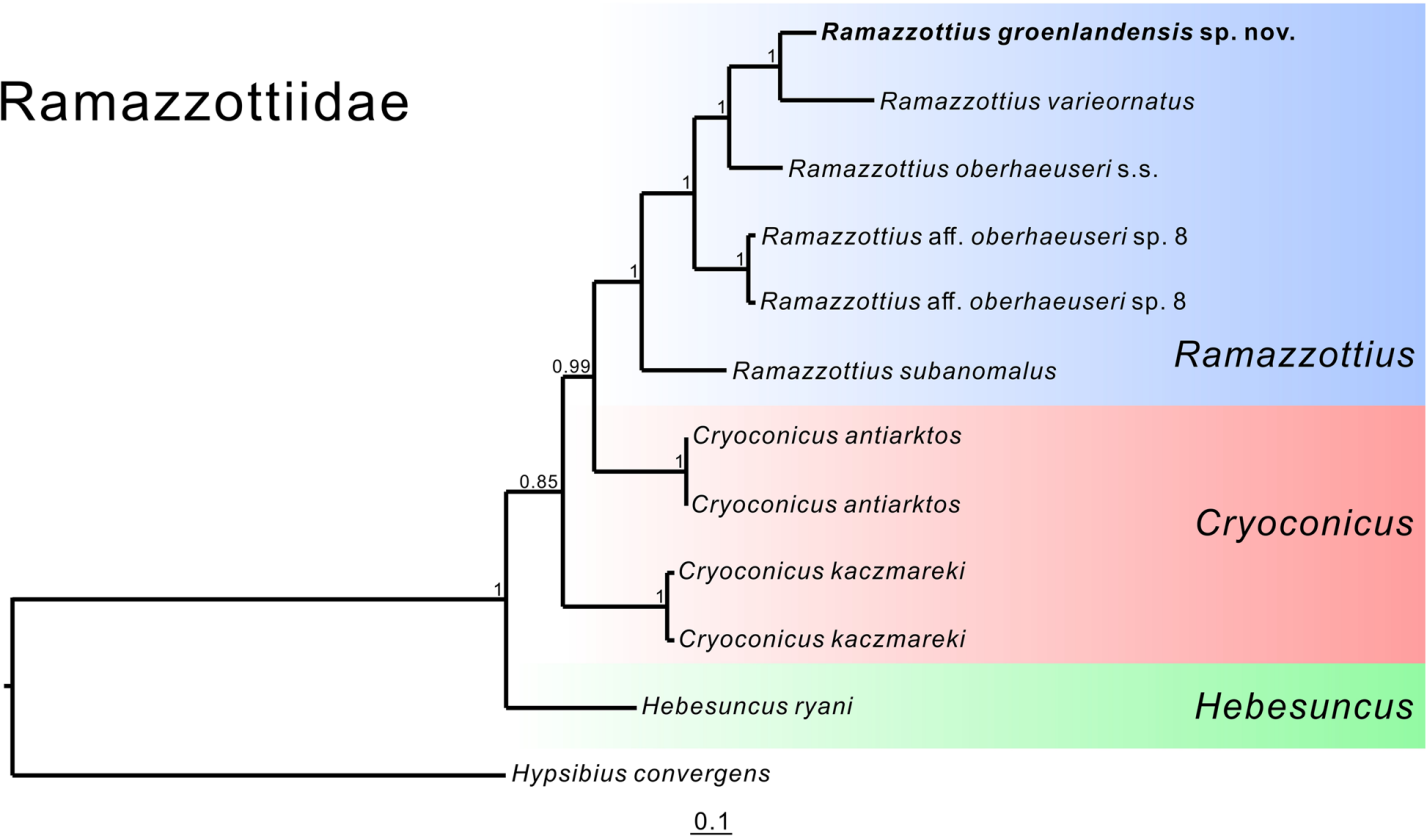
A concatenated 18S rRNA + 28S rRNA + COI consensus tree based on the Bayesian Inference (BI) analysis, with *Hypsibius convergens* as the outgroup. Node values are given as BI posterior probability (PP) values

The concatenated 18S rRNA + 28rRNA + COI phylogenetic reconstruction based on the Bayesian inference analysis shows a stable topology in the family Ramazzottiidae (Fig. 7), in which *Ramazzottius* g*roenlandensis* sp. nov. is positioned as a sister group of *R. varieornatus*.

**Fig. 8:**
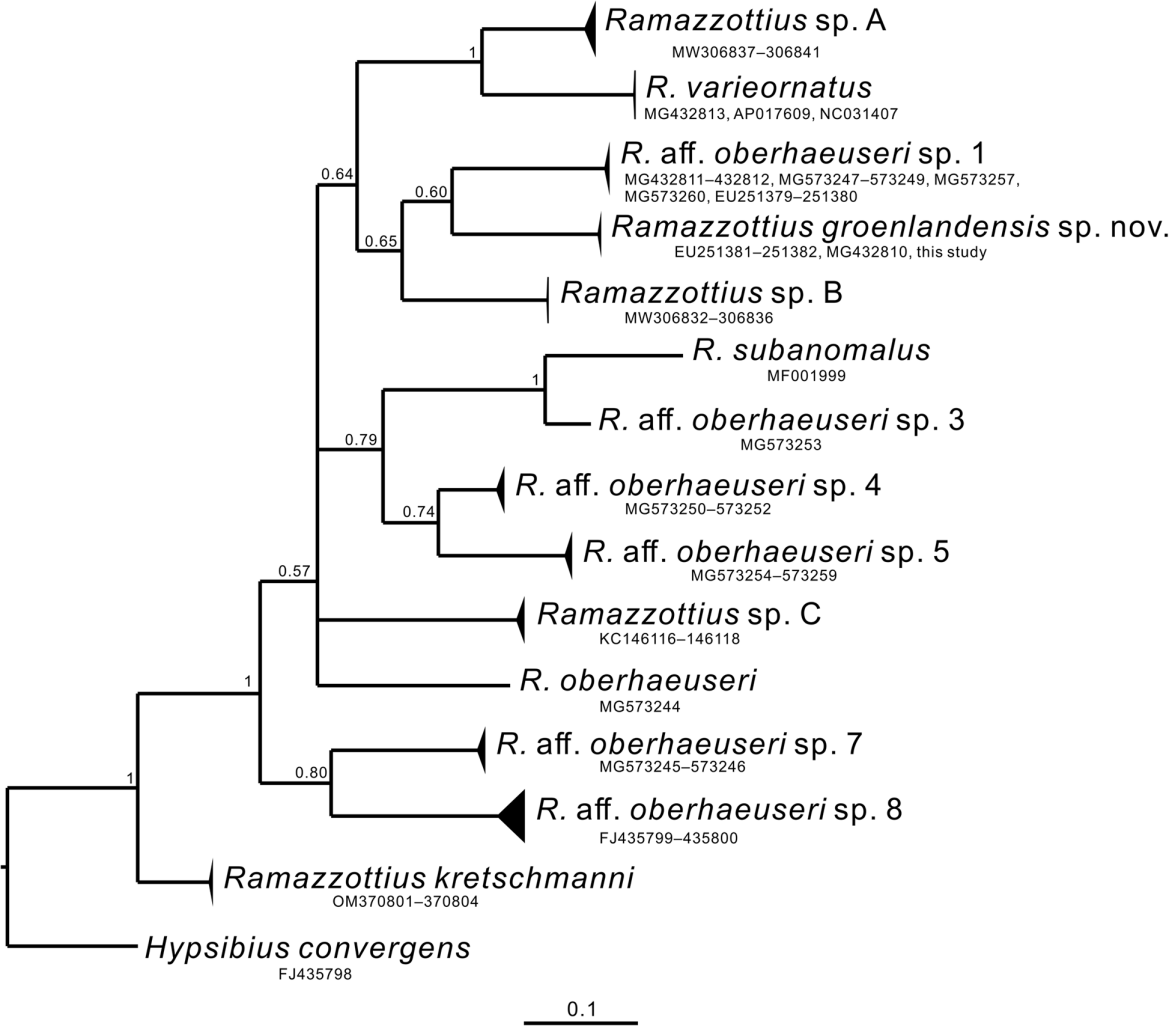
A Bayesian inference (BI) phylogenetic tree constructed using COI sequences of the genus *Ramazzottius*. Among the molecular species *R*. aff. *oberhaeuseri* species 1–8 identified by Stec et al. [31], *R*. aff. *oberhaeuseri* species 2 is revealed as *R*. *groenlandensis* sp nov. (as explained in the text). Node values mean BI posterior probability values. Scale bar represents substitution per position

The COI phylogenetic reconstruction based on Bayesian inference analysis also confirms that *Ramazzottius* cf. *rupeus* (MG432810) [37] and *Ramazzottius* cf. *oberhaeuseri* species 2 (EU251381–2) in fact belong to *R. groenlandensis* sp. nov (Fig. 8).

### Remarks

Several ramazzottiid species, including *Ramazzottius groenlandensis* sp. nov., have a papilla on the lateral part of the leg IV [31, 35, 37]. The presence of this organ has been suggested to be sexually dimorphic, being present only in males [53, 55, 75, 76]. In *R*. *groenlandensis* sp. nov., 39% of observed specimens showed a papilla on the lateral part of the leg IV. The papilla on leg IV varies in size (Fig. 5C–E); from a quarter of leg IV to more than half of the length of the leg when the leg is fully extended (Fig. 5D). It may reflect the physiological condition of each specimen, or potential deformation during the preparation process.

Due to the similar adult morphology and the lack of detailed descriptions in early reports, egg traits are used as common and the key taxonomic characters in *Ramazzottius* [31, 36, 38]. However, many *Ramazzottius* specimens have been reported without eggs, e.g., *R. edmondabouti* [52], *R. szeptycki* [59], *R. semisculptus* [77], *R. belubellus* [51], or *R. thulini* [54].

Additionally, a prominent intraspecific variation warns against the description of eggs based on a few specimens [78]. Particularly in the genus *Ramazzottius*, considerable intraspecific variation in the morphology of the egg processes has been documented from several species: e.g. *R. kretschmanni* Guidetti, Cesari, Giovannini, Ebel, Forschler & Schill, 2022 [53], *R. littoreus*, *R. oberhaeuseri* [31], *R. sabatiniae*, and *R. subanomalus* (Biserov, 1985) [36, 75]. Therefore, both SEM and DIC pictures and measurements of eggs should be provided in the description of species belonging to the genus *Ramazzottius*.

## Discussion

### Homology of head sensory organs

The full set of head sensory organs of *Ramazzottius groenlandensis* sp. nov., recognized under SEM, is most likely homologous to the sensory organs of heterotardigrades. Some eutardigrade species have one or two pairs of head sensory organs [21, 30, 32, 37, 40, 79, 80], while most eutardigrades possess specific innervated areas on the head surface, called the sensory fields—the circumoral sensory field (COS), the anterolateral sensory field (ASF), the ventrolateral sensory field (VSF), and the posterolateral sensory field (PSF) [22, 23, 81]—which are not clearly visible under DIC observation. *Ramazzottius* g*roenlandensis* sp. nov. has paired sensory organs on the surface of the head, i.e., the frontal lobes, the anteroventral lobes (AVL), and the elliptical organs (EO) (Fig. 3). Based on their relative positions, the sensory organs of *R. groenlandensis* sp. nov. are compared to the sensory fields of other eutardigrades: frontal lobes vs. ASF; AVL vs. VSF; EO vs. PSF (Table 3). Frontal lobes and elliptical organs have been observed in several eutardigrades. Some isohypsibioid tardigrades, such as *Ursulinius pappi* (Iharos, 1966) [82] and *Apodibius confusus* Dastych, 1983 [83] (see Fig. 4B & E of [32]), possess frontal lobes. The papilla cephalica (= cephalic papilla [34]) of *Halobiotus* [80] is also likely to be homologous to the frontal lobes of *R. groenlandensis* sp. nov. Elliptical organs have been found in some hypsibioids (*Calohypsibius*, *Cryoconicus*, and *Ramazzottius*) [40] and isohypsibioids (*Fractonotus*) [30]. The position, innervation from the brain, and shape of the temporalia in *Halobiotus* can also be compared to the elliptical organ [21]. Apochelan tardigrades have the cephalic papillae, which can be related to AVL of *R. groenlandensis* sp. nov. [22]. Despite their distinct morphologies, similar innervation patterns may imply homology between the head sensory organs of heterotardigrades and the head sensory fields of eutardigrades [22, 23, 34, 84, 85]. Particularly, Gross et al. [20] suggested the homology of head sensory organs and head sensory fields between heterotardigrades and eutardigrades based on immunohistochemical data. Head sensory organs of *R. groenlandensis* sp. nov. possess micropores (Fig. 3), which suggests that these structures could function as mechano-chemoreceptors, as do the cirri and clavae of heterotardigrades [19].

**Fig. 9:**
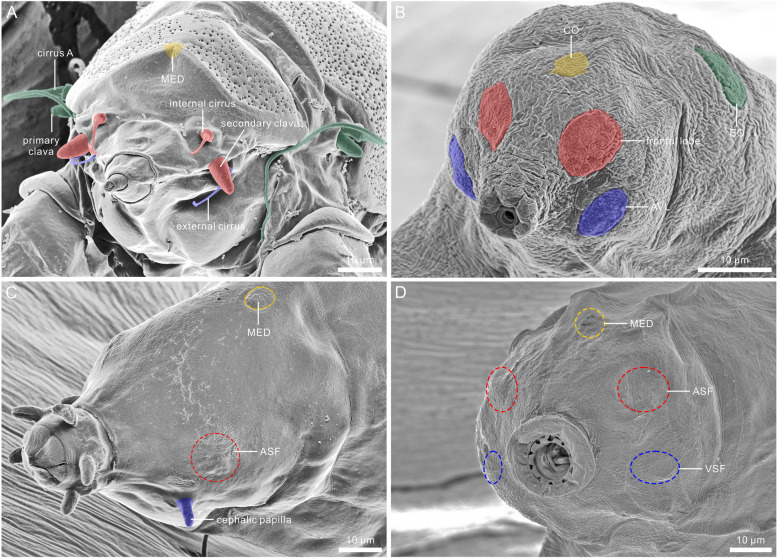
False-colored scanning electron microscopic images of heterotardigrades and eutardigrades. This figure follows the hypothesis for the homology of head sensory organs and head sensory fields by Gross et al. [20]. **A** heterotardigrade *Echiniscus testudo*. **B** eutardigrade *Ramazzottius groenlandensis* sp. nov. **C** eutardigrade *Milnesium* sp. **D** eutardigrade *Paramacrobiotus areolatus*. Colored area and dotted area mean head sensory organs and head sensory fields, respectively. Red: internal cirri & secondary clavae / frontal lobes / anterolateral sensory field (ASF). Blue: external cirri / anteroventral lobes (AVL) / cephalic papillae / ventrolateral sensory field (VSF). Yellow: median sensory field (MED) / centrodorsal organ (CO). Green: cirrus A & primary clava / elliptical organ (EO) / posterolateral sensory field (PSF)

The centrodorsal organ (CO), has a small pore at its center (Fig. 3A, C). A few eutardigrades, such as *Doryphoribius dawkinsi* Michalczyk & Kaczmarek, 2010 [86] and *Ursulinius pappi* [32], also possess a CO. The position, different morphology of the cuticle, and the small pore in the centre of this structure are significantly similar to those of the limnoterrestrial echiniscoidean *Echiniscus testudo* (Doyère, 1840) [24] structure, which is considered to be homologous to the unpaired median cirrus of marine heterotardigrades [20]. Thus, *Ramazzottius groenlandensis* sp. nov. displays a set of head sensory organs that is most probably identical in origin, position, and function to that of heterotardigrades, i.e., frontal lobes vs. a pair of internal cirri + secondary clavae; AVL vs. a pair of external cirri; EO vs. a pair of lateral cirri + primary clavae; CO vs. unpaired median cirrus (Fig. 9; Table 3). Our findings strongly support the hypothesis that head sensory organs in eutardigrades are homologous to those in heterotardigrades, corroborating the homology between head sensory organs of heterotardigrades and sensory fields of eutardigrades [20].

**Table 3:** Homology of the head sensory structures in the heterotardigrade species *Echiniscus testudo* and eutardigrade species *Ramazzottius groenlandensis* sp. nov. and *Hypsibius exemplaris*, based on Gross et al. [20]

*Echiniscus testudo* (heterotardigrade)	*Ramazzottius groenlandensis* sp. nov. (eutardigrade)	*Hypsibius exemplaris* (eutardigrade)
Internal cirri + secondary clavae	Frontal lobe	Anterolateral sensory field (ASF)
External cirri	Anteroventral lobe (AVL)	Ventrolateral sensory field (VSF)
Lateral cirri + primary clavae	Elliptical organ (EO)	Posterolateral sensory field (PSF)
Median sensory field (MED) (Median cirrus in marine heterotardigrades)	Centrodorsal organ (CO)	Median sensory field (MED)

### *Ramazzottius groenlandensis* sp. nov. and *Ramazzottius oberhaeuseri* species complex

The detailed integrative redescription [31] of *Ramazzottius oberhaeuseri* established the delimitation criterion of both *R. oberhaeuseri sensu stricto* and the *R. oberhaeuseri* species complex: *R. oberhaeuseri* species complex was defined as a cluster of *Ramazzottius* species characterized by eggs with hemispherical processes. In addition to *R. thulini* and *R. libycus*, the phylogenetic analysis using COI sequences of [31] identified eight potential species within the complex (*R*. aff. *oberhaeuseri* sp. 6 was resolved as *R*. *oberhaeuseri sensu stricto*). However, based on the COI sequence data from this study, *Ramazzottius* cf. *oberhaeuseri* species 2 from [31] has been reclassified as *Ramazzottius groenlandensis* sp. nov. Since the eggs of *R. groenlandensis* sp. nov. possess conical processes rather than hemispherical processes, this suggests that *R. groenlandensis* sp. nov. is not a member of the *R. oberhaeuseri* species complex.

Although posterior probability (pp) values appear to be low, the COI-based phylogenetic analysis conducted in this study (Fig. 8) presents nine potential *Ramazzottius* species, a conclusion further supported by the results obtained from pairwise genetic distance calculations and ASAP analysis (Supplementary Data 3). Within these nine species, six species of the *R. oberhaeuseri* complex (*R*. aff. *oberhaeuseri* species 1, 3–5, 7, 8) as identified in the previous study [31] are also recovered. Notably, despite the modest pp values, the tree suggests that (*R*. aff. *oberhaeuseri* species 7 + *R*. aff. *oberhaeuseri* species 8) forms a sister group to all other *Ramazzottius* species, excluding *R. kretschmanni*. Moreover, the 18S + 28S + COI phylogenetic analysis (Fig. 7) also indicates that *R*. aff. *oberhaeuseri* species 8 forms a group with (*R. oberhaeuseri* + *R. varieornatus* + *R. groenlandensis* sp. nov.). Therefore, *R. oberhaeuseri* species complex is not supported phylogenetically. This implies that *R. oberhaeuseri* group is likely to be a morphogroup, rather than a species complex.

### Distribution of *Ramazzottius groenlandensis* sp. nov.

*Ramazzottius groenlandensis* sp. nov. displays a notably extensive distribution range spanning from Greenland and Svalbard to Italy, with the most distant localities being approximately 4,000 km apart. However, it appears that *R. groenlandensis* sp. nov. predominantly occupies specific environments. Notably, the three localities where the species was found are characterized by polar and mountainous conditions, strongly suggesting a preference for cold environments. This inclination aligns with similar tendencies observed in other several tardigrade lineages, such as *Cornechiniscus holmeni*, *Bertolanius*, *Macrobiotus ariekammensis*, and *Cryoconicus* [87–90]. Recently, an experiment has proposed phoresis, particularly involving snails, as a means of short-distance terrestrial tardigrade dispersal [91]. However, the mechanism by which terrestrial tardigrades can disperse over such long distances remains unresolved, as both birds and wind have been proposed as possible dispersal vectors [90].

## Conclusion

The integrative description of *Ramazzottius groenlandensis* sp. nov. provided insights into the evolution of the head sensory organs within tardigrades. The correspondence of seven head structures of *R. groenlandensis* sp. nov. to the cephalic cirri and clavae of heterotardigrades supports the homology of head sensory organs between heterotardigrades and eutardigrades. This result suggests that the last common ancestor of Eutardigrada could have possessed the set of sensory organs on the head. Furthermore, the results of molecular analyses imply that the *R. oberhaeuseri* group is likely to be a morphogroup, rather than a species complex.

### Supplementary Information

**Additional file 1:**  **Supplementary Fig. 1.** The study area of this study: Ella Island.

** Additional file 2:**  **Supplementary Table 1.** Primer information for genotyping.

** Additional file 3:**  **Supplementary Table 2.** PCR condition for this study.

** Additional file 4:**  **Supplementary Data 1.** GenBank accession numbers of the 18S, 28S, and COI sequences used for the 18S + 28S + COI phylogenetic analysis of this study (see Fig. 7).

** Additional file 5:**  **Supplementary Data 2.** GenBank accession numbers of the COI sequences used for the COI phylogenetic analysis of this study (see Fig. 8).

** Additional file 6:**  **Supplementary Data 3.** Pairwise genetic distances of COI, 18S, and 28S, and ASAP result.

** Additional file 7:**  **Supplementary Data 4.** Raw measurement data of *Ramazzottius groenlandensis* sp. nov.

## Data Availability

The datasets used and/or analysed during the current study are available from the corresponding author on reasonable request. This article has been registered at Zoobank (https://zoobank.org/5F851FDE-7017-4244-A257-46AF8FD983AA).
