# Supplementary figures and images for: Homology of the head sensory structures between Heterotardigrada and Eutardigrada supported in a new species of water bear (Ramazzottiidae: Ramazzottius)
Source: Zoological Lett. 2023 Nov 27;9:22. doi: 10.1186/s40851-023-00221-w (PMC10680360; doi:10.1186/s40851-023-00221-w)

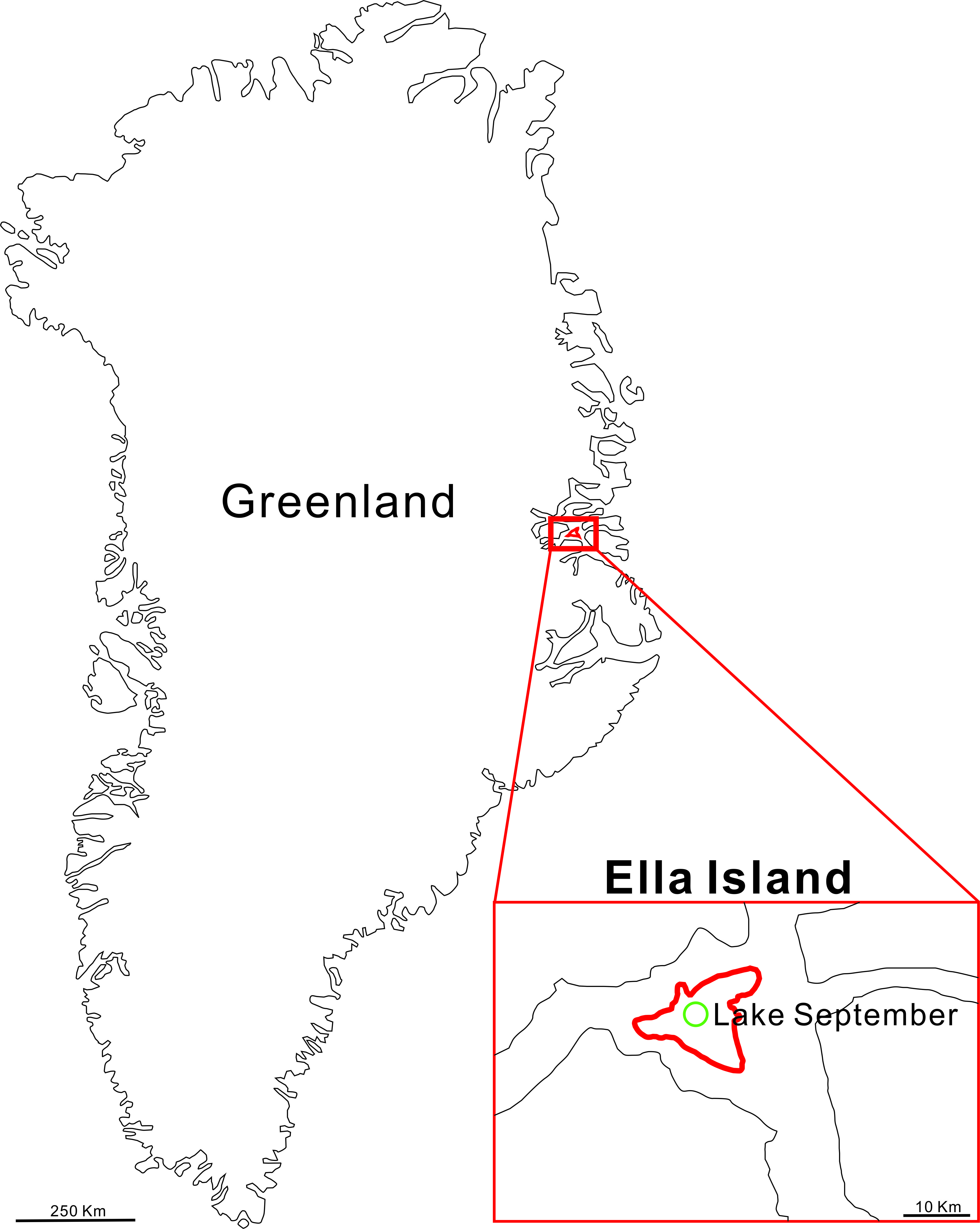

Supplement: Supplementary file 1 — Additional file 1: Supplementary Fig. 1. The study area of this study: Ella Island. [file 40851_2023_221_MOESM1_ESM.jpg]
