# Supplementary material for: Homology of the head sensory structures between Heterotardigrada and Eutardigrada supported in a new species of water bear (Ramazzottiidae: Ramazzottius)
Source: Zoological Lett. 2023 Nov 27;9:22. doi: 10.1186/s40851-023-00221-w (PMC10680360; doi:10.1186/s40851-023-00221-w)
Supplement: Supplementary file 3 — Additional file 3: Supplementary Table 2. PCR condition for this study. [file 40851_2023_221_MOESM3_ESM.pdf]

DNA fragment	Primer name	Primer direction	Primer sequence (5'-3')	Source
COI	LCO1490	Forward	GGTCAACAAATCATAAAGATATTG	Folmer et al. (1994)
	HCO2198	Reverse	TAAACTTCAGGGTCACCAAAAAATCA	
18S rRNA	SSU01_F	Forward	AACCTGGTTGATCCTGCCAGT	Sands et al. (2008)
	SSU82_R	Reverse	TGATCCTTCTGCAGGTTCACCTAC	
28S rRNA	28SF0001	Forward	ACCCVCYNAATTTAAGCATAT	Mironov et al. (2012)
	28SR0990	Reverse	CCTTGGTCCGTGTTTCAAGAC	
