# Supplementary material for: Homology of the head sensory structures between Heterotardigrada and Eutardigrada supported in a new species of water bear (Ramazzottiidae: Ramazzottius)
Source: Zoological Lett. 2023 Nov 27;9:22. doi: 10.1186/s40851-023-00221-w (PMC10680360; doi:10.1186/s40851-023-00221-w)
Supplement: Supplementary file 4 — Additional file 4: Supplementary Data 1. GenBank accession numbers of the 18S, 28S, and COI sequences used for the 18S + 28S + COI phylogenetic analysis of this study (see Fig. 7). [file 40851_2023_221_MOESM4_ESM.pdf]

Step	COI			18S			28S		
	Temperature °C	Time [min:sec]	Cycles	Temperature °C	Time [min:sec]	Cycles	Temperature °C	Time [min:sec]	Cycles
Initial denaturation	94	05:00	1	94	05:00	1	95	05:00	1
Denatureation	94	01:00	5	–	–	–	–	–	–
Annealing	45	01:30	5	–	–	–	–	–	–
Elongation	72	01:30	5	–	–	–	–	–	–
Denaturation	94	01:00	35	94	00:45	35	95	00:30	35
Annealing	50	01:30	35	60	01:30	35	50	01:30	35
Elongation	72	01:00	35	72	02:00	35	72	01:00	35
Final elongation	72	05:00	1	72	10:00	1	72	05:00	1
Source	Michalczyk et al. 2012			Zeller 2010			Mironov et al. 2012		
